# Help-Seeking Behavior and Psychological Distress by Age in a Nationally Representative Sample of Japanese Employees

**DOI:** 10.2188/jea.JE20190042

**Published:** 2020-06-05

**Authors:** Takashi Yamauchi, Machi Suka, Hiroyuki Yanagisawa

**Affiliations:** Department of Public Health and Environmental Medicine, The Jikei University School of Medicine, Tokyo, Japan

**Keywords:** help-seeking behavior, mental health, workers, occupational health, representativeness, Japan

## Abstract

**Background:**

The present study aimed to examine the association between the presence/absence of help-seeking behavior (ie, behavior aimed at obtaining assistance from others to improve a situation or problem) and psychological distress among private and public employees by age group using a nationally representative sample of the Japanese population.

**Methods:**

The present study analyzed data obtained from the 2016 Comprehensive Survey of Living Conditions, a nationwide cross-sectional survey. Of 568,426 participants, 78,284 private and public employees aged 20 to 59 years, who were receiving no mental health services at the time of the survey and reported at least one stressor in daily life, were eligible. The primary outcome measure was self-rated psychological distress as measured by the Kessler Psychological Distress Scale. Multiple logistic regression analyses were carried out separately by age group, adjusting for sociodemographic and job/life-related factors.

**Results:**

The proportion of participants not showing help-seeking behavior was significantly higher among those aged 40–59 years compared to those aged 20–39 (30.5% and 22.7%, respectively; *P* < 0.001). Participants without help-seeking behavior had significantly higher odds ratios (ORs) for psychological distress, regardless of age group (OR = 1.9 [95% confidence interval (CI), 1.6–2.0] and OR = 1.6 [95% CI, 1.4–1.7] for the age 20–39 years and 40–59 years groups, respectively), compared to those showing help-seeking behavior.

**Conclusions:**

Participants not showing help-seeking behavior were more likely to have severe psychological distress, and this trend appeared to be slightly stronger among those aged 20–39 years. These findings suggest that promoting help-seeking behavior is important for improving mental health among workers.

## INTRODUCTION

Prevention of severe psychological distress and mental disorders through early intervention and the promotion of help-seeking has been a major public health issue. Previous systematic reviews have suggested that several factors, such as low mental health literacy, financial issues, and stigma and negative attitudes towards people with mental health problems, influence help-seeking behavior (ie, behavior aimed at obtaining assistance from others to improve a situation or problem) for psychological distress or mental health-related problems.^1^^,^^2^

Promoting help-seeking behavior for mental health problems among workers has also been an important occupational health issue worldwide. Previous studies regarding help-seeking behavior for mental health issues among workers have focused on specific industries or occupations, such as military personnel,^3^ medical professionals,^4^ ambulance personnel,^5^ railway workers,^6^ police workers,^7^ and firefighters,^8^ and all have used unrepresentative data. To our knowledge, no previous study has examined associations between help-seeking behavior and mental health issues among workers in various industries/occupations using nationally representative data.

Previous research has shown that the types of or resources for help-seeking behavior for mental health problems vary across age groups. Compared to older persons, young individuals tend to avoid seeking professional help for mental health issues and use more informal sources of help, such as family members, parents, and friends.^9^^,^^10^ Thus, it is necessary to examine age-specific associations between help-seeking behavior and mental health issues among workers.

Since 1986, the Japanese Ministry of Health, Labour and Welfare (MHLW) has conducted the Comprehensive Survey of Living Conditions (CSLC), which provides a large database of information on Japanese residents’ basic living conditions, including working conditions, occupation/job-type, mental health status, and help-seeking behavior.^11^ The CSLC is a cross-sectional nationwide survey using a representative sample of the Japanese population. It is one of Japan’s designated Fundamental Statistics.

Thus, the present study examined the association between the presence/absence of help-seeking behavior and psychological distress among private and public employees by age group, using a nationally representative sample of the Japanese population from the CSLC. A better understanding of the age-specific associations between help-seeking behavior and psychological distress using nationally representative data may contribute to promoting help-seeking behavior for mental health issues among workers with different ages and demographic/job-related backgrounds.

## METHODS

### Data source

The present study analyzed data obtained from the CSLC.^11^ The large-scale CSLC has been conducted by the MHLW every 3 years to investigate basic living conditions in order to develop and implement a national policy regarding health, labor, and welfare. The CSLC comprises various questionnaires and is addressed to all members of households selected randomly from national census districts in Japan.

In the present study, we analyzed data from the Household Questionnaire and the Health Questionnaire of the 2016 CSLC, which included 224,208 households with 568,426 individuals (response rate, 77.5%).^11^

**Figure 1.**
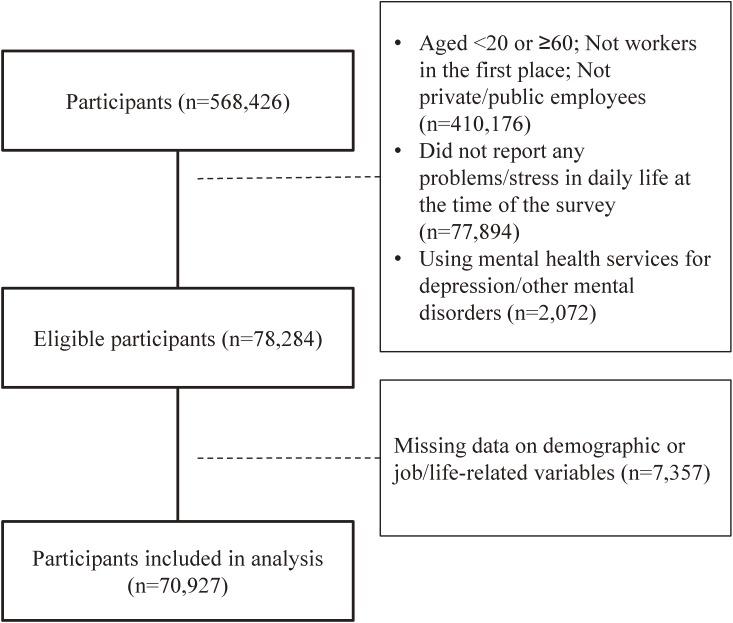
Participant selection

The eligibility criteria for the study participants were as follows: (1) Japanese residents aged 20 to 59 years, (2) private or public employees (ie, not a homemaker or student), (3) having reported at least one stressor in daily life, and (4) having received no mental health services for depression or other mental disorders at the time of the survey. In the CSLC Health Questionnaire, a single question asked participants whether they were private or public employees, regardless of contract type. In addition, only participants who reported at least one stressor answered questions concerning help-seeking behavior; thus, participants who did not report any stressor in daily life were excluded. Furthermore, to examine the association between help-seeking behavior and mental distress, we removed the participants who were receiving psychiatric treatment and medication. With regard to the presence/absence of mental health service use, participants were asked the question “Are you attending a hospital due to illnesses/injuries at present?” Those who answered “yes” were subsequently asked to select all illnesses/injuries from a list of 42 categories, including depression and/or other mental disorders. Of 568,426 participants, 78,284 were eligible for the present study (Figure 1).

Ethics approval was not required for the current study based on the “Ethical Guidelines for Medical and Health Research involving Human Subjects” by the Japanese Government,^12^ as this study involved a secondary analysis of national surveillance data, which did not include any personally identifiable information. De-identified individual-level data from the CSLC are available for scientific research following approval by the MHLW under Article 33 of Japan’s Statistics Act.^13^

### Measures

The primary independent variable in the present study was presence/absence of help-seeking behavior regarding problems and stress in daily life. In the CSLC Health Questionnaire, participants were asked the question “Do you have any problems or stress in your daily life at present?” Those who answered “yes” were subsequently asked to select all possible causes of their problems/stress from a list of 21 categories, including relationship with family members, love and sex, marriage, divorce, bullying/harassment, and job. Furthermore, they were asked whether they were consulting someone from a list of eight categories (family members, friends/colleagues, supervisors, counseling services in public organizations, counseling services in private organizations, physicians at hospitals/clinics, counseling spaces within the media, and other) regarding their present problems/stressors. Participants who reported not seeking any help were asked to select their reasons from the following three options: (1) “I want to consult, but I have not done so yet,” (2) “I want to consult, but I do not know where to consult,” and (3) “I do not need to consult anyone.” Participants were asked to select all that applied from among the 11 categories.

As potential confounders, other demographic (sex, age [20–39 or 40–59 years], marital status [married, never married, and widowed/divorced]), job-related (working hours per week [≤39 h, 40–49 h, 50–59 h, and ≥60 h], job type, type of employment contract [standard (full-time) or non-standard (including part-time)], and presence/absence of job-related stress), and life-related variables (presence/absence of stress other than job, presence/absence of habitual heavy drinking [either <300 or ≥300 g ethanol/week] and smoking [current smoker or never/past smoker], and hours of sleep over the past month [<5 h, 5–7 h, and ≥7 h]) were also measured. Based on the Japan Standard Occupational Classification established by the Japanese Ministry of Internal Affairs and Communications,^14^ job type was categorized into the following seven subtypes: managerial, professional/technical, clerical, sales/service, manufacturing, transport/construction, and other workers.

The primary outcome measure for the present study was self-rated psychological distress. Non-specific psychological distress during the last 30 days was measured with the Kessler Psychological Distress Scale (K6).^15^ The K6 consists of six items that assess psychological distress over the previous 30 days (ie, “How often did you feel nervous?,” “How often did you feel hopeless?,” “How often did you feel restless or fidgety?,” “How often did you feel so depressed that nothing could cheer you up?,” “How often did you feel that everything was an effort?,” and “How often did you feel worthless?”). Respondents rate each item on a five-point scale (0 = none of the time, 1 = a little of the time, 2 = some of the time, 3 = most of the time, 4 = all of the time). Total scores thus range from 0 to 24, with higher scores indicating higher psychological distress. Following previous studies, we defined a K6 score of ≥13 as indicating severe psychological distress. In the present study, we used the Japanese version of the K6, which has high internal consistency and reliability.^16^

### Statistical analysis

First, for each variable, cross-tabulation was conducted by age group. To examine whether the frequency distribution of the variables, including help-seeking, differed by age group, we performed chi-square and Fisher’s exact tests. To examine whether the frequency distribution of the type of help-seeking differed by the presence/absence of psychological distress and age group, we performed Fisher’s exact tests.

To examine the association between the presence/absence of help-seeking behavior and psychological distress, logistic regression analyses were carried out with the presence/absence of severe psychological distress as the dependent variable. In the logistic regression models, we adjusted for demographic factors (ie, sex and marital status), job-related factors (ie, working hours per week, job type, employment contract, and job-related stress), and life-related factors (ie, non-job-related stress, sleep hours, and drinking/smoking habit). The presence/absence of job/non-job-related stress was assessed using a list of 21 categories of stressors, and stressors other than those related to a job were recategorized as “non-job-related stress.” To examine whether the association between the presence/absence of help-seeking behavior and psychological distress differed by age group, logistic regression analyses were carried out by age group.

*P* < 0.05 was considered statistically significant. All analyses were conducted using SPSS version 25 (IBM, Chicago, IL, USA).

## RESULTS

Of the 78,284 eligible participants, 7,357 had missing data on demographic and job/life-related variables. These participants’ data were removed from the study. Thus, data from 70,927 participants (33,714 females and 37,213 males; mean age, 41.6 [standard deviation, 10.5] years) were included in the analysis (Figure 1).

Table 1 shows the distribution of demographic and job/life-related data, including help-seeking behavior, by age group. Except for smoking status, there were significant differences in the distribution of each variable by age group. The proportion of participants with severe psychological distress was significantly higher among those aged 20–39 years compared to those aged 40–59 years (9.0% and 6.0%, respectively; *P* < 0.001).

**Table 1.** Frequency distribution of variables by age group

	20–39 years		40–59 years		Chi-square test/Fisher’s exact test	Effect size
	(*n* = 29,258)		(*n* = 41,669)			
	
	*n*	(%)	*n*	(%)		
**Sex**					*P* < 0.001	0.02
Male	14,961	(51.1)	22,252	(53.4)		
Female	14,297	(48.9)	19,417	(46.6)		
**Marital status**					*P* < 0.001	0.39
Married	12,794	(43.7)	30,277	(72.7)		
Never married	15,230	(52.1)	6,608	(15.9)		
Widowed/divorced	1,234	(4.2)	4,784	(11.5)		
**Working hours per week**					*P* < 0.001	0.02
≤39 h	5,675	(19.4)	8,680	(20.8)		
40–49 h	14,240	(48.7)	20,316	(48.8)		
50–59 h	5,488	(18.8)	7,639	(18.3)		
≥60 h	3,855	(13.2)	5,034	(12.1)		
**Job type**					*P* < 0.001	0.15
Managerial workers	607	(2.1)	3,478	(8.3)		
Professional/technical workers	9,489	(32.4)	11,874	(28.5)		
Clerical workers	5,321	(18.2)	7,822	(18.8)		
Sales/service workers	7,433	(25.4)	8,714	(20.9)		
Manufacturing workers	3,180	(10.9)	4,278	(10.3)		
Transport/construction workers	1,249	(4.3)	2,418	(5.8)		
Other workers	1,979	(6.8)	3,085	(7.4)		
**Employment contract**					*P* < 0.001	0.01
Standard	22,403	(76.6)	31,393	(75.3)		
Non-standard	6,855	(23.4)	10,276	(24.7)		
**Job-related stress**					*P* < 0.001	0.07
Present	19,653	(67.2)	25,165	(60.4)		
Absent	9,605	(32.8)	16,504	(39.6)		
**Other stress**					*P* < 0.001	0.06
Present	21,889	(74.8)	33,134	(79.5)		
Absent	7,369	(25.2)	8,535	(20.5)		
**Sleep hours**					*P* < 0.001	0.10
<5 h	2,618	(8.9)	5,463	(13.1)		
5–7 h	20,842	(71.2)	30,406	(73.0)		
≥7 h	5,798	(19.8)	5,800	(13.9)		
**Heavy drinking**					*P* < 0.001	0.10
Present	1,987	(6.8)	5,370	(12.9)		
Absent	27,271	(93.2)	36,299	(87.1)		
**Smoking status**					*P* = 0.90	0.00
Current smoker	7,706	(26.3)	10,956	(26.3)		
Never/past smoker	21,552	(73.7)	30,713	(73.7)		
**Help-seeking**					*P* < 0.001	0.09
Present	22,616	(77.3)	28,947	(69.5)		
Absent	6,642	(22.7)	12,722	(30.5)		
**K6 score**					*P* < 0.001	0.06
≥13	2,619	(9.0)	2,519	(6.0)		
<13	26,639	(91.0)	39,150	(94.0)		

K6, Kessler Psychological Distress Scale.

Table 2 shows the association between the type of help-seeking and the presence/absence of psychological distress by age group. The proportion of participants without help-seeking behavior was significantly higher among those aged 40–59 compared to those aged 20–39 (30.5% and 22.7%, respectively; *P* < 0.001). Regardless of age group, the proportion of participants without help-seeking behavior was significantly higher among those with severe psychological distress than those without (33.9% vs 21.6% and 40.1% vs 29.9% for ages 20–39 and 40–59, respectively; both *P* < 0.001). In both age groups, family members, friends, and supervisors in the workplace were the most frequently reported sources of help sought by respondents.

**Table 2.** Help-seeking and Kessler Psychological Distress Scale (K6) score by age group

	20–39 years								40–59 years								Fisher’s exact test (total vs total)	Effect size
	
	Total		K6 ≥13		K6 <13		Fisher’s exact test	Effect size	Total		K6 ≥13		K6 <13		Fisher’s exact test	Effect size		
	(*N* = 29,258)		(*n* = 2,619)		(*n* = 26,639)				(*N* = 41,669)		(*n* = 2,519)		(*n* = 39,150)					
					
	*n*	(%)	*n*	(%)	*n*	(%)			*n*	(%)	*n*	(%)	*n*	(%)				
**Any help-seeking behavior**	22,616	(77.3)	1,732	(66.1)	20,884	(78.4)	*P* < 0.001	0.09	28,947	(69.5)	1,508	(59.9)	27,439	(70.1)	*P* < 0.001	0.05	*P* < 0.001	0.09
Family members	16,127	(55.1)	1,153	(44.0)	14,974	(56.2)	*P* < 0.001	0.07	20,428	(49.0)	977	(38.8)	19,451	(49.7)	*P* < 0.001	0.05	*P* < 0.001	0.06
Friends/colleagues	15,733	(53.8)	1,192	(45.5)	14,541	(54.6)	*P* < 0.001	0.05	16,792	(40.3)	881	(35.0)	15,911	(40.6)	*P* < 0.001	0.03	*P* < 0.001	0.13
Supervisors	5,072	(17.3)	380	(14.5)	4,692	(17.6)	*P* < 0.001	0.02	4,717	(11.3)	234	(9.3)	4,483	(11.5)	*P* = 0.001	0.02	*P* < 0.001	0.09
Counseling services in public organizations	272	(0.9)	50	(1.9)	222	(0.8)	*P* < 0.001	0.03	763	(1.8)	71	(2.8)	692	(1.8)	*P* < 0.001	0.02	*P* < 0.001	0.04
Counseling services in private organizations	108	(0.4)	29	(1.1)	79	(0.3)	*P* < 0.001	0.04	291	(0.7)	34	(1.3)	257	(0.7)	*P* < 0.001	0.03	*P* < 0.001	0.02
Physicians at a hospital/clinic	746	(2.5)	101	(3.9)	645	(2.4)	*P* < 0.001	0.03	2,385	(5.7)	171	(6.8)	2,214	(5.7)	*P* = 0.02	0.01	*P* < 0.001	0.08
Counseling spaces on the media	182	(0.6)	25	(1.0)	157	(0.6)	*P* = 0.03	0.01	276	(0.7)	24	(1.0)	252	(0.6)	*P* = 0.07	0.01	*P* = 0.54	0.00
Other	326	(1.1)	44	(1.7)	282	(1.1)	*P* = 0.005	0.02	659	(1.6)	53	(2.1)	606	(1.5)	*P* = 0.04	0.01	*P* < 0.001	0.02
**No help-seeking behavior**	6,642	(22.7)	887	(33.9)	5,755	(21.6)	*P* < 0.001	0.09	12,722	(30.5)	1,011	(40.1)	11,711	(29.9)	*P* < 0.001	0.05	*P* < 0.001	0.09
“I want to consult, but I have not done so yet”	1,211	(4.1)	359	(13.7)	852	(3.2)	*P* < 0.001	0.15	2,483	(6.0)	502	(19.9)	1,981	(5.1)	*P* < 0.001	0.15	*P* < 0.001	0.04
“I want to consult, but I do not know where to consult”	720	(2.5)	210	(8.0)	510	(1.9)	*P* < 0.001	0.11	1,279	(3.1)	259	(10.3)	1,020	(2.6)	*P* < 0.001	0.11	*P* < 0.001	0.02
“I do not need to consult anyone”	4,785	(16.4)	436	(16.6)	4,349	(16.3)	*P* = 0.68	0.00	9,093	(21.8)	398	(15.8)	8,695	(22.2)	*P* < 0.001	0.04	*P* < 0.001	0.07

Table 3 shows the results of logistic regression analyses using presence/absence of psychological distress as the dependent variable by age group. After adjusting for sociodemographic and job/life-related variables, participants without help-seeking behavior had significantly higher odds ratios (ORs) for psychological distress, regardless of age group (OR 1.86; 95% confidence interval [CI], 1.6–2.0 and OR 1.56; 95% CI, 1.4–1.7 for ages 20–39 and 40–59, respectively), compared with those reporting help-seeking behavior. For both age groups, the OR for psychological distress was significantly higher for those with non-job-related stress (ie, stress due to factors other than job-related factors) and those sleeping less than 5 h per day.

**Table 3.** Logistic regression analyses with Kessler Psychological Distress Scale (K6) score as dependent variable by age group

Age group	20–39 years (*n* = 29,258)				40–59 years (*n* = 41,669)			
	
	Univariate		Multivariate		Univariate		Multivariate	
			
	Odds ratio	(95% CI)	Odds ratio	(95% CI)	Odds ratio	(95% CI)	Odds ratio	(95% CI)
**Sex**								
Male	1.00	(0.9–1.1)	0.95	(0.8–1.0)	0.89	(0.8–1.0)	0.89	(0.8–1.0)
Female	(ref)		(ref)		(ref)		(ref)	
**Marital status**								
Married	(ref)		(ref)		(ref)		(ref)	
Never married	1.60	(1.4–1.7)	1.57	(1.4–1.7)	1.49	(1.3–1.6)	1.32	(1.1–1.5)
Widowed/divorced	1.54	(1.2–1.9)	1.33	(1.0–1.6)	1.56	(1.3–1.8)	1.32	(1.1–1.5)
**Working hours per week**								
≤39 h	(ref)		(ref)		(ref)		(ref)	
40–49 h	0.85	(0.7–0.9)	0.87	(0.7–1.0)	0.88	(0.7–1.0)	0.94	(0.8–1.1)
50–59 h	0.85	(0.7–1.0)	0.88	(0.7–1.0)	0.83	(0.7–0.9)	0.90	(0.7–1.0)
≥60 h	1.07	(0.9–1.2)	1.08	(0.9–1.3)	1.10	(0.9–1.3)	1.09	(0.9–1.3)
**Job type**								
Managerial workers	0.87	(0.6–1.2)	0.94	(0.6–1.3)	0.69	(0.5–0.8)	0.76	(0.6–0.9)
Professional/technical workers	0.96	(0.8–1.1)	0.97	(0.8–1.1)	0.84	(0.7–0.9)	0.86	(0.7–1.0)
Clerical workers	(ref)		(ref)		(ref)		(ref)	
Sales/service workers	1.19	(1.0–1.4)	1.10	(0.9–1.2)	1.03	(0.9–1.2)	0.98	(0.8–1.1)
Manufacturing workers	1.25	(1.0–1.5)	1.20	(1.0–1.4)	1.05	(0.9–1.2)	1.02	(0.8–1.2)
Transport/construction workers	0.90	(0.7–1.1)	0.90	(0.7–1.1)	0.87	(0.7–1.1)	0.83	(0.6–1.0)
Other workers	1.17	(0.9–1.4)	1.08	(0.8–1.3)	1.13	(0.9–1.3)	1.08	(0.9–1.3)
**Employment contract**								
Standard	(ref)		(ref)		(ref)		(ref)	
Non-standard	1.35	(1.2–1.5)	1.22	(1.0–1.4)	1.26	(1.1–1.4)	1.15	(1.0–1.3)
**Job-related stress**								
Present	1.56	(1.4–1.7)	2.21	(2.0–2.4)	1.61	(1.4–1.8)	2.13	(1.9–2.3)
Absent	(ref)		(ref)		(ref)		(ref)	
**Other stress**								
Present	2.35	(2.0–2.6)	2.99	(2.6–3.4)	2.10	(1.8–2.4)	2.62	(2.2–3.0)
Absent	(ref)		(ref)		(ref)		(ref)	
**Sleep hours**								
<5 h	2.74	(2.3–3.2)	2.41	(2.0–2.8)	3.34	(2.8–3.9)	2.99	(2.5–3.5)
5–7 h	1.27	(1.1–1.4)	1.24	(1.1–1.4)	1.21	(1.0–1.4)	1.17	(1.0–1.3)
≥7 h	(ref)		(ref)		(ref)		(ref)	
**Heavy drinking**								
Present	1.11	(0.9–1.3)	1.25	(1.0–1.5)	1.25	(1.1–1.4)	1.38	(1.2–1.6)
Absent	(ref)		(ref)		(ref)		(ref)	
**Smoking status**								
Current smoker	1.09	(0.9–1.2)	1.06	(0.9–1.2)	1.15	(1.0–1.3)	1.11	(1.0–1.2)
Never/past smoker	(ref)		(ref)		(ref)		(ref)	
**Help-seeking**								
Present	(ref)		(ref)		(ref)		(ref)	
Absent	1.86	(1.7–2.0)	1.86	(1.6–2.0)	1.57	(1.4–1.7)	1.56	(1.4–1.7)

CI, confidence interval.

## DISCUSSION

In the present study, we examined the association between the presence/absence of help-seeking behavior and psychological distress by age group in a nationally representative sample of Japanese workers. The proportion of participants without help-seeking behavior was significantly higher among older participants compared to younger participants. Participants without help-seeking behavior tended to have severe psychological distress regardless of age group, and this trend appeared to be slightly stronger among those aged 20–39 years.

Among the 2016 CSLC participants not using any mental health services for depression or other mental disorders, 9% and 6% of those aged 20–39 and 40–59 years, respectively, reported severe psychological distress. Previous studies in Japan reported that approximately 4% of the general public aged 18 or older had severe psychological distress defined by a K6 score of ≥13 in 2007, 2010, 2013, and 2016.^17^ These differences in the proportions of those with severe psychological distress might be partially due to the fact that the sample in the present study consisted only of private/public employees with problems in their daily lives who did not use mental health services.

The proportion of participants with severe psychological distress was significantly higher among younger participants compared to older participants. These findings are consistent with previous studies showing that the proportion of individuals with severe psychological distress was higher among young people. These trends have gained strength in recent years in economically developed countries, including Japan.^17^^,^^18^ Furthermore, the results of the present study might be consistent with the suicide statistics from the National Police Agency of Japan showing that the proportion of suicides due to work-related issues appeared to be higher among those aged 20–29 years than among older people.^19^

The proportion of participants without help-seeking behavior was significantly higher among older participants compared to younger participants. Mental health literacy, defined as knowledge and understanding regarding symptoms and treatment of mental health issues, was found to be one of the key factors for help-seeking behavior related to mental health issues such as depression.^20^^,^^21^ The findings of the current study are consistent with previous studies suggesting that younger individuals have better mental health literacy than older persons. Moreover, poor mental health literacy may delay help-seeking.^22^

After adjusting for sociodemographic and job/life-related variables, participants without help-seeking behavior were more likely to have severe psychological distress regardless of age group. Help-seeking behavior has been suggested to have a buffer effect against psychological distress. Our findings suggest that removing barriers to help-seeking behavior, especially among those who did not have help resources (eg, family members living in the same house, close friends/colleagues, and/or familiar supervisors) is crucial for improving mental health among workers.

The present study analyzed the association between the presence/absence of help-seeking behavior and psychological distress among workers adjusting for relevant job/life-related factors, using a large representative data in Japan with a moderate response rate. However, this study has several limitations. First, although the CSLC is a large nationwide survey using a representative sample, due to its cross-sectional nature it was not possible to determine causality between help-seeking behavior and psychological distress. For instance, intention to seek help may be partially affected by the mental health status of participants, and vice versa. Second, psychological distress, the dependent variable, was assessed by self-ratings rather than using structured interviews with mental health professionals. However, the K6 has been validated in studies using a diagnostic structured interview method.^23^ Third, there was no information concerning the industries (ie, job areas) of the CSLC participants. However, the CSLC included questions regarding participants’ occupation/job-type, and this factor was adjusted for as a potential confounder in the analyses in the present study. Finally, caution should be exercised when generalizing the present findings to populations with different backgrounds, as the sample in the present study was restricted to private and public employees in Japan in 2016.

### Conclusions

The proportion of participants without help-seeking behavior was significantly higher among older participants compared to younger participants. After adjusting for sociodemographic and job/life-related variables, participants without help-seeking behavior were more likely to have severe psychological distress regardless of age group. This trend appeared to be slightly stronger among those aged 20–39 years. The findings of this study using a nationally representative sample of the Japanese population suggest that, regardless of age, promoting help-seeking behavior is important for improving mental health among workers.
